# Decreasing brain activity caused by acute administration of ketamine and alcohol – A randomized, controlled, observer-blinded experimental study

**DOI:** 10.3389/fphar.2024.1456009

**Published:** 2024-10-16

**Authors:** Luan Oliveira Ferreira, Esther Padilha da Silveira, Clarissa A. Paz, Maria K. Otake Hamoy, Gabriela B. Barbosa, Murilo F. Santos, Raína M. Conceição, Anthony Lucas G. Amaral, Karina Dias Resende, Dielly Catrina Favacho Lopes, Moisés Hamoy

**Affiliations:** ^1^ Laboratory of Experimental Neuropathology, João de Barros Barreto University Hospital, Federal University of Pará, Belém, Brazil; ^2^ Department of Anesthesiology, João de Barros Barreto University Hospital, Federal University of Pará, Belém, Brazil; ^3^ Laboratory of Pharmacology and Toxicology of Natural Products, Biological Science Institute, Federal University of Pará, Belém, Brazil

**Keywords:** ketamine, alcohol-related disorders, electroencephalography, brain waves, substance-related disorders

## Abstract

**Introduction:**

Substance abuse is a major public health problem. In recent years, ketamine, which is a parenteral anesthetic, has been consumed increasingly as an illicit drug together with alcohol, although little is known of how this association alters brain activity. The present study investigated the influence of progressive doses of ketamine, associated with alcohol, on electrophysiological activity.

**Methods:**

For this, 72 late-adolescent (8–10-week-old) male Wistar rats received either ketamine only, at low (10 mg/kg), intermediate (20 mg/kg) or high (30 mg/kg) doses via intraperitoneal injection, or alcohol (2 mL/100 g) via oral gavage followed by ketamine (at low, intermediate, and high doses). Electroencephalograms (EEG) and electromyographic recordings were obtained 5 min after the final application of the drug.

**Results:**

When administered alone, ketamine resulted in an increase in delta, theta, beta, and gamma brainwaves, with a more pronounced effect being detected at the highest dose (30 mg/kg) in the case of the delta, beta, and gamma waves. The amplitude of the alpha brainwaves was reduced at all doses of ketamine, but less intensively at the highest dose. When administered alone, alcohol reduced all the brainwaves, with the reduction in the alpha waves being exacerbated by ketamine at all doses, and that of the theta and beta waves being boosted at the lowest dose. The intermediate dose of ketamine (20 mg/kg) reverted the alcohol-induced reduction in the theta and gamma waves, whereas the high dose increased delta, theta, beta, and gamma bandpower.

**Discussion:**

Overall, then, while ketamine enhances the depressant effects of alcohol on the alpha brainwave at all doses, a low dose intensified this effect on the theta and beta 175 waves, whereas a high dose produces neuronal hyperexcitability in the theta and 176 gamma bandpower.

## 1 Introduction

Recreational substance abuse has a long, and ongoing history (Olsen, 2022). The consumption of psychoactive drugs is currently one of the principal concerns of the World Health Organization (WHO), due not only to its toxicological and social effects, but also because of the complexity of the potential long-term effects of these drugs. Worse still, the combined use of psychoactive ^3^ substances has become a common practice in young people, primarily in the 15–30-year age group (The United Nations Office on Drugs and Crime, 2024).

Alcohol, a depressant of the Central Nervous System (CNS), is the world’s most widely consumed intoxicant (Kuntsche et al., 2017). The detrimental effects of alcohol are often either downplayed or obscured by conflicting information on the harmful or beneficial effects of this substance. Social norms that support drinking behavior, together with the commercial value of the alcohol industry, may also offset attempts to adopt a healthy lifestyle or reduce the burden of alcoholism on public health systems (World Health Organization, 2024).

Alcohol is widely used as a recreational intoxicant, and is linked to almost 10% of deaths, worldwide, through traffic accidents, crime, vandalism, and chronic health problems such as cirrhosis. Binge drinking is the most frequent pattern of alcohol consumption in adolescents and young adults, and is common at social events over weekends, where young people are encouraged to drink by peer pressure (Ivaniushina and Titkova, 2021). Binge drinking tends to provoke acute changes in behavior, such as extroversion, impulsiveness, and sensation-seeking, and has been linked to psychiatric disorders such as stress, anxiety, trauma, and depression (Kuntsche et al., 2017). Binge drinking has differential impacts on the different developmental stages of the organism. For example, Brancato et al. (2018) found that binge-like alcohol consumption in the pre-conceptional and peri-gestational periods induced depression in the offspring of rats, which may persist in the adolescent (Brancato et al., 2018). Constant repetition of binge drinking is known to impair decision-making and is associated with unsafe sexual behavior in young social drinkers, which indicates that excessive alcohol has adverse effects on the prefrontal neural systems associated with reflection impulsivity (Townshend et al., 2014). In addition to being a CNS depressant and acting through several different mechanisms, the ingestion of alcohol is frequently associated with the consumption of other substances, such as cocaine, lysergic acid diethylamide (LSD) and, more recently, ketamine (Marco et al., 2022). While alcohol has, historically, been considered to be the gateway to other drugs, this role has been filled increasingly by marijuana, which now tends to be the first drug in the progression toward polysubstance abuse in adolescents (Keyes et al., 2019; Kandel and Kandel, 2015).

Ketamine is a parenteral anesthetic with analgesic, amnesic, and hypnotic effects, which provides a good safety margin in terms of respiratory depression (Gao et al., 2016). It is a classical, non-competitive antagonist of the N-methyl-D-aspartate (NMDA) receptors, and acts through GABAergic interneurons, which provoke the disinhibition of pyramidal activity in the cerebral cortex (Sleigh et al., 2014). Recent data have revealed the increasing non-therapeutic use of ketamine, primarily as a recreational drug, being referred to as “Key”, “Special K” or “Kit Kat” in English-speaking countries (Lee et al., 2022). This recreational use is often associated with the consumption of other substances, such as alcohol, which is becoming increasingly common, especially among teenagers and young adults, who frequent bars and other gatherings, such as house parties, where the ketamine may often be administered by unconventional means, such as nasal inhalation or smoking, in addition to the traditional intravenous and oral routes (Lee et al., 2022; Krystal et al., 2013).

It is important to note here that the psychoactive effects of both alcohol and ketamine share a number of pathways in the CNS and may often have equivalent effects on the same receptor (MacDonald et al., 1987). Like ketamine, for example, alcohol inhibits NMDA receptors in a non-competitive way (Peoples et al., 1997). Research has also shown that the acute, concomitant use of ketamine and alcohol may induce cognitive behavioral disorders, such as depression and memory deficits, by inducing apoptosis in the cells of the prefrontal cortex (Li et al., 2019). The abuse of ketamine may thus cause cognitive retardation, visual problems, schizophrenic symptoms, and damage to the cerebellum (Li et al., 2019; Liu et al., 2017). These effects are doubly preoccupying, given the potential for addiction from this combination of drugs, due to its activation of dopaminergic effects on the neurons in the ventral tegmental area (Zhang et al., 2018).

In addition to the synergistic effects associated with the pharmacological mechanisms in the glutamatergic, noradrenergic, and cholinergic pathways, the combination of alcohol and ketamine involves pharmacokinetical interactions through the CYP3A4 hepatic enzyme, given that binge drinking saturates the metabolic enzymes that reduce the metabolization of the ketamine and increases its bioavailability (Kamp et al., 2020; Kunitoh et al., 1993). Given this, alcohol enhances the toxicological effects of the ketamine, which results in hepatobiliary, respiratory, cardiac, and urinary malfunction, and accounts for the changes in consciousness, cognition, and emotionality provoked by the association of these drugs (Kobayashi et al., 2022).

Even so, little is known about the acute changes in the brain connections provoked by the association of these two substances in the brain. While a number of studies of the behavioral impairment caused by the association of alcohol with ketamine, such as schizophrenic symptoms, depression, anxiety, and cognitive deficit, it is unclear whether alcohol modifies the electrical activity of the brain when associated with ketamine, and how these changes are manifested at different doses of this drug (Kobayashi et al., 2022). In this context, the present study used electroencephalogram (EEG) recordings to investigate the cerebral oscillations associated with the acute administration of ketamine, either alone or in combination with alcohol, in late-adolescent Wistar rats. By providing data on the functional state of the brain linked to perceptual, cognitive, and emotional functions, EEG is a fundamental tool for the monitoring of shifts in neuronal activity associated with exposure to drugs, and a translational animal model is appropriate for the evaluation of disorders provoked by substance abuse (Spencer, 2014; Blume, 2006).

## 2 Materials and methods

### 2.1 Study animals and the experimental design

The present study was approved by the Ethics Committee for laboratory research on animals of the Federal University of Pará in Belém, Brazil (protocol 3396040822). All the data reported here were also collected in compliance with the ARRIVE (Animal Research: Reporting *In Vivo* Experiments) guidelines (Percie du Sert et al., 2020). All necessary precautions were taken to prevent animal suffering and distress. All the procedures reported here were conducted strictly between 08:00 a.m. and 10:00 a.m.

For the present study, 72 male Wistar rats (8–10 weeks old, with a mean weight of 240 ± 20 g) were obtained from the Central Animal Facility of the Biological Sciences Institute of the Federal University of Pará (ICBUFPA) in Belém, northern Brazil. This age group was selected based on the temporal profile of postnatal rat brain maturation, given that the available evidence (Mengler et al., 2014) indicates that developmental changes in brain volume continue until 3 months (12 weeks) of age. The rats selected for the present study can thus be classified as late adolescent or young adult individuals. The animals were housed in polypropylene cages covered with a metallic grid in a controlled environment (mean temperature of 22°C ± 2°C; 12/12 h light/dark cycle) with *ad libitum* access to standard rat chow and water.

**FIGURE 1:**
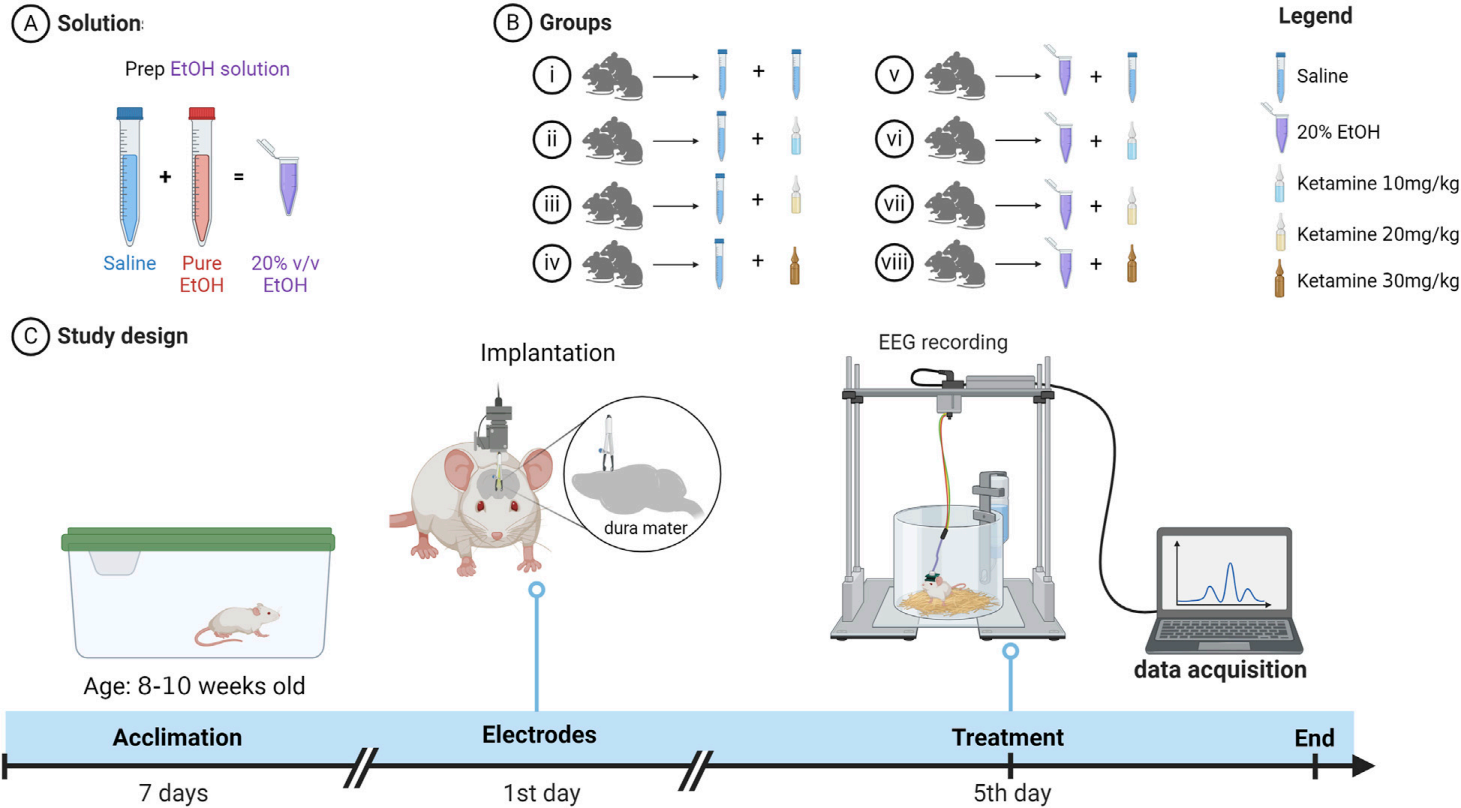
Pictorial diagram showing **(A)** the preparation of the solution, **(B)** the allocation of the eight treatment groups, and **(C)** the timeline of the experiment. EEG = Electroencephalography.

The experimental design of the present study is summarized in Figure 1. After a 7-day acclimation period, electrodes were implanted in the region of the somatosensory cortex of each rat, and 4 days later the animals were allocated randomly to one of eight groups (n = 9 animals/group): (i) animals that received saline via oral gavage and, after 15 min, 0.9% saline intraperitoneally (i.p.); (ii) saline via oral gavage and, after 15 min, ketamine 10 mg/kg i.p.; (iii) saline via oral gavage and, after 15 min, ketamine 20 mg/kg i.p.; (iv) saline via oral gavage and, after 15 min, ketamine 30 mg/kg i.p.; (v) animals that received alcohol at a dose of 2 mL/100 g (20% v/v alcohol solution) via oral gavage and, after 15 min, 0.9% saline i.p.; (vi) alcohol (2 mL/100 g) via oral gavage and, after 15 min, ketamine (10 mg/kg) i.p.; (vii) alcohol (2 mL/100 g) via oral gavage and, after 15 min, ketamine (20 mg/kg) i.p., and (viii) alcohol (2 mL/100 g) via oral gavage and, after 15 min, ketamine (30 mg/kg) i.p.

The recording of electroencephalographic activity was initiated 5 min after the intraperitoneal administration of the saline or ketamine and lasted for 3 min. Once the EEG data had been recorded, the tail of the animal was tagged with a group-coded tag. The solutions used in the study were stored in bottles of identical appearance, which were labelled randomly by a researcher who was not involved in the study.

### 2.2 Drugs

Five different chemical substances were employed in the present study and were obtained from different sources. The anesthetic ketamine hydrochloride was obtained from the König Laboratory in Santana de Parnaíba, in São Paulo state, Brazil, while xylazine hydrochloride was sourced from the Vallée Laboratory in Montes Claros, Minas Gerais, Brazil. Lidocaine, a local anesthetic without vasoconstrictor, was obtained from the Hipolabor Laboratory in Sabará, Minas Gerais. Ethyl alcohol (P.A.), for dilution at a concentration of 20%, was sourced from Sigma-Aldrich, St. Louis, Missouri, United States, and ketoprofen from Ceva Saúde Animal Ltda in Paulínia, São Paulo state, Brazil.

### 2.3 Electroencephalography

The animals were sedated using an intraperitoneal injection of xylazine hydrochloride (10 mg/kg). Following the abolition of the interdigital reflex, the animals were placed in a stereotaxic apparatus, for the rapid surgical exposure of the skull. Stainless-steel electrodes that had been sterilized in an autoclave were placed on the dura mater at bregma coordinates −0.96 mm and ±1.0 mm laterally and fixed with self-curing acrylic cement. The animals were then transferred to individual cages and treated for 3 days with ketoprofen (5 mg/kg, administered subcutaneously) for the control of postoperative pain (CEUA, 2022).

On the fifth day after surgery, the electrodes were connected to a digital data acquisition system composed of a high impedance amplifier (Grass technologies, P511), an oscilloscope (Protek, 6510), and a board for the acquisition and digitization of the data (National Instruments, Austin, TX). The animals were confined in acrylic boxes of restrictive size (20 cm × 45 cm x 15 cm) for the continuous collection of the data. A standard EEG protocol was employed for all the different treatments, with 5 min of accommodation being followed by a 3-min period of data recording.

The offline analysis used a tool created in the Python programming language (version 2.7). The “Numpy” and “Scipy” libraries were used for mathematical processing, and the “matplolib” library for producing the plots. A graphical user interface was developed using the PyQt4 library. The spectrograms were compiled using a Hamming window with 256 points (256/1000 s). Each frame of the Power Spectral Density (PSD) was generated with an overlap of 128 points per window. The PSD was calculated for each frame by Welch’s mean periodogram method. Frequency histograms were compiled by calculating the PSD of the signal using a 256-point non-overlapping Hamming window, which yielded a resolution of 1 Hz per box. The mean PSDs were calculated for each group and are shown here in individual boxes. The analyses were run at a frequency of up to 40 Hz and divided into five bands for the interpretation of the brainwave patterns in the different treatments, following the classification of Ferreira et al. (2020): delta (1–4 Hz), theta (4–8 Hz), alpha (8–12 Hz), beta (12–28 Hz) and gamma (28–40 Hz).

### 2.4 Electromyography

Two electrodes (2 mm in length, diameter of 0.2 mm) were conjugated at 2 mm to determine the respiratory rate of the animals and measure the power of their muscle contractions. These electrodes were inserted into the 10th intercostal space to record muscle activity, with the data being collected over a 1-min interval.

The contractive power of the intercostal muscle was determined by the potential difference between the electrodes, with the spectral power being calculated within the range of up to 40 Hz. The frequency of contraction of the intercostal muscle was interpreted as a measure of the breathing movement of the animal, and was thus used to calculate its respiratory rate, in breaths per minute.

### 2.5 Euthanasia

Following the collection of the data, the animals were euthanized with an overdose of ketamine hydrochloride (100 mg/kg i.p.) and xylazine (10 mg/kg i.p.), to avoid suffering. These procedures were in accordance with the Brazilian National Council for the Control of Animal Experimentation and the Ethics Committee on Use of Animals of the Biological Sciences Institute at the Federal University of Pará requirements for euthanasia.

### 2.6 Statistical analysis

The normality and homogeneity of the variation in the data were verified using the Kolmogorov-Smirnov test. When the residuals were distributed normally and the variances were homogeneous, the groups were compared using a one-way ANOVA followed by Tukey’s *post hoc* test for pairwise comparisons. When these assumptions were not met, the data were verified using the Kruskal–Wallis non-parametric analysis of variance followed by Dunn’s *post hoc* test for pairwise comparisons. The data are presented as mean values followed by their respective standard deviations, i.e., mean ± SD. The data were analyzed in GraphPad Prism, version 9 (Graph-Pad Software Inc., San Diego, CA, United States), and a *p* < 0.05 significance level was considered for all the procedures.

## 3 Results

### 3.1 Ketamine induces greater irregularity in the EEG trace and the power distribution of higher frequency brainwaves than alcohol alone or in combination

**FIGURE 2:**
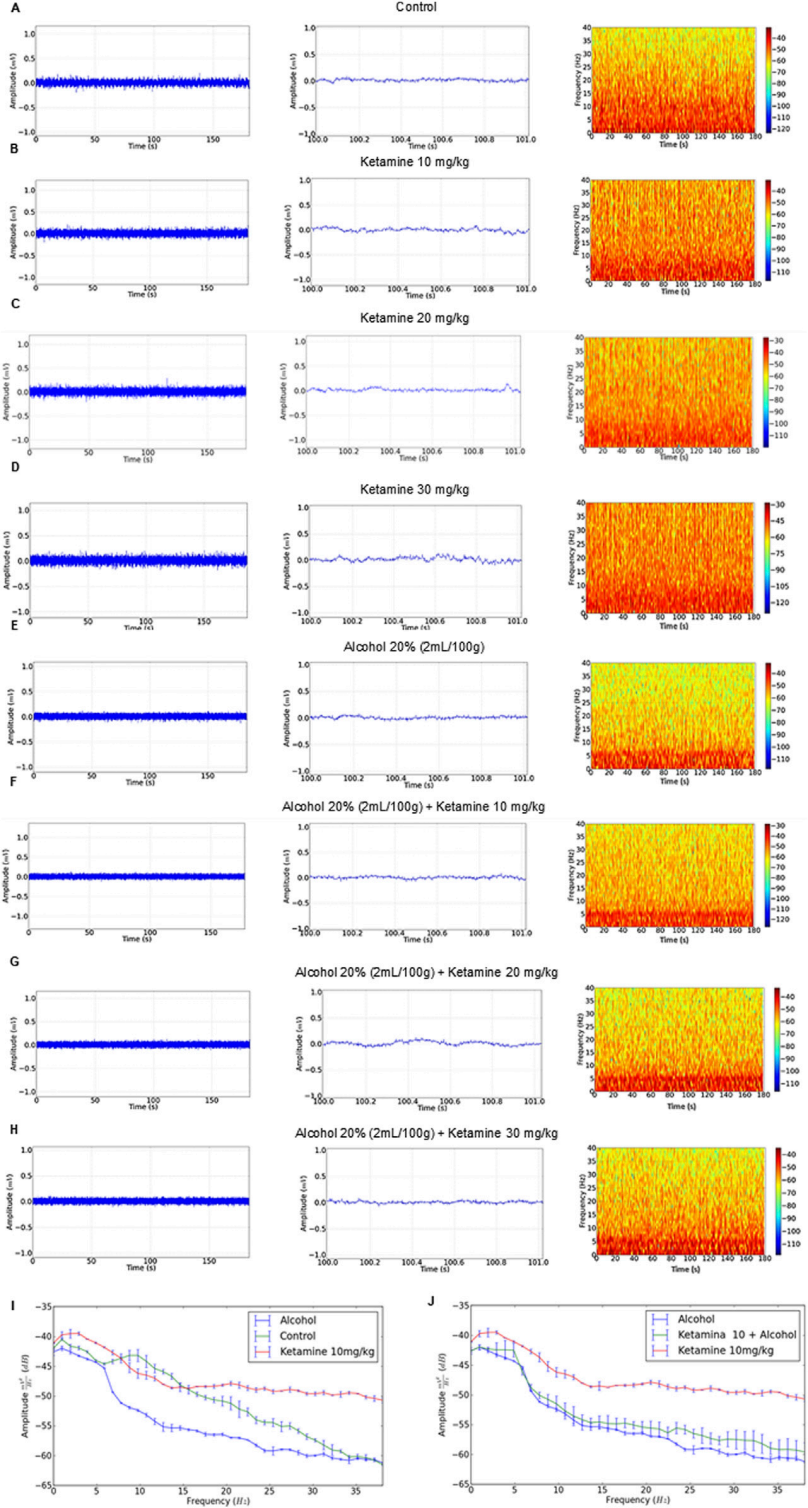
Electroencephalographic traces recorded over 3 min (left), with a representative 1-s fragment (center), and frequency spectrogram (right) obtained from a single representative animal in each of the eight treatment groups: **(A)** Control; **(B)** Ketamine 10 mg/kg; **(C)** Ketamine 20 mg/kg; **(D)** Ketamine 30 mg/kg; **(E)** Alcohol; **(F)** Alcohol + ketamine 10 mg/kg; **(G)** Alcohol + ketamine 20 mg/kg; **(H)** Alcohol + ketamine 30 mg/kg.

The EEG recordings of the control group revealed a regular trace with an amplitude of less than 0.1 mV (Figure 2A, left), as observed in the amplification of 1 s (Figure 2A, center), and a greater concentration of energy below 15 Hz (Figure 2A, right). The amplitude of the traces of the ketamine treatment groups (at all doses – 10, 20, and 30 mg/kg) were all close to 0.1 mV (Figures 2B–D, left), with irregularity in the 1-s amplification, increasing with increasing doses (Figures 2B–D, center). These animals also presented greater power distribution at 40 Hz, with a greater intensity at frequencies below 10 Hz on the spectrogram (Figures 2B–D, right).

In the case of the alcohol only group, on the other hand, the EEG readings had a mean wave amplitude of 0.06 mV (Figure 2E, left and center), with greater energy intensity below 5 Hz (Figure 2E, right). The combination of ketamine with alcohol presented patterns similar to the alcohol only group, with traces at an amplitude of below 0.1 mV and the greatest concentration of energy below 5 Hz (Figures 2F–H).

### 3.2 Ketamine alters the spectral power of the brainwaves in a dose-dependent manner

**FIGURE 3:**
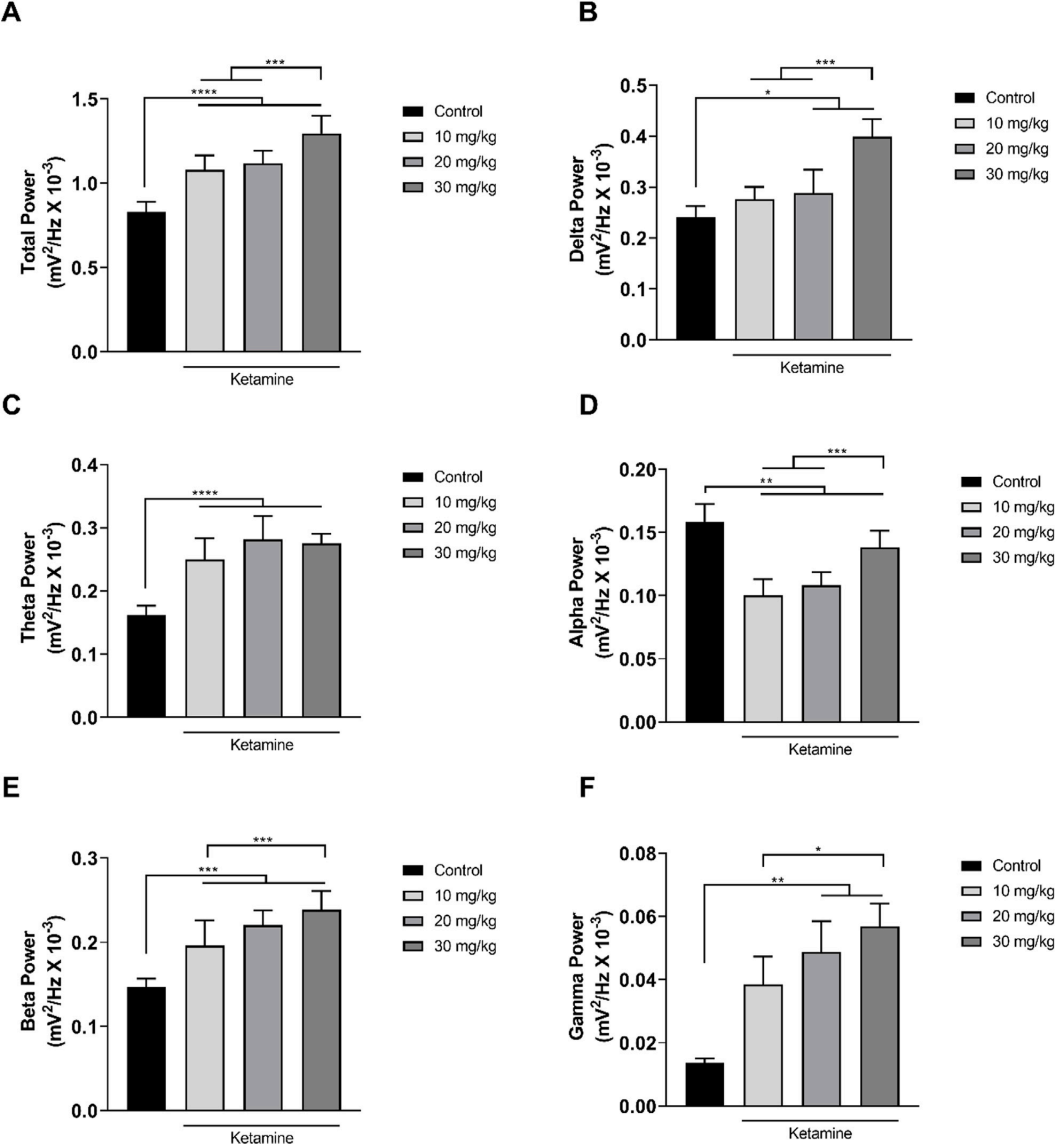
Linear distribution of the frequencies (mean ± SD) of the brainwaves of the animals in the ketamine and control groups (*n* = 9 animals/group): **(A)** Total bandpower; **(B)** Delta wave bandpower; **(C)** Theta wave bandpower; **(D)** Alpha wave bandpower; **(E)** Beta wave bandpower; **(F)** Gamma wave bandpower. **(A–E)**: one-way ANOVA with Tukey’s *post hoc* test. **(F)** Kruskal–Wallis test with Dunn’s *post hoc* test. (*p < 0.05, **p < 0.01, ***p < 0.001, ****p < 0.0001).

In the analysis of the distribution of the linear frequencies of up to 40 Hz (Figure 3A; Table 1), a total wave power of 0.83 ± 0.06 mV^2^/Hz x 10^−3^ was recorded for the control group. Significantly higher total power was recorded in all the ketamine groups in comparison with control (*p* < 0.0001 in all cases), and the total power of the highest dose ketamine group (30 mg/kg) was also significantly higher than that of the two lower-dose groups (*p* < 0.001 in both cases).

**TABLE 1:** Electroencephalographic parameters of the total bandpower and the power of the delta, theta, alpha, beta, and gamma brainwaves in the animals exposed to different doses of ketamine.

Mean ± SD total power (mV^2^/Hz x 10^-3^): F_(3, 32)_ = 47.60, p < 0.0001			
Control	Ketamine at a dose of:		
	10 mg/kg	20 mg/kg	30 mg/kg
0.83 ± 0.06	1.08* ± 0.085	1.17* ± 0.08	1.295*^# ± 0.10

Mean ± SD delta power (mV^2^/Hz x 10^-3^): F_(3, 32)_ = 39.17, p < 0.0001			
Control	Ketamine at a dose of:		
	10 mg/kg	20 mg/kg	30 mg/kg
0.24 ± 0.02	0.29 ± 0.02	0.29* ± 0.045	0.4*^# ± 0.03

Mean ± SD theta power (mV^2^/Hz x 10^-3^): F_(3, 32)_ = 37.79, p < 0.0001			
Control	Ketamine at a dose of:		
	10 mg/kg	20 mg/kg	30 mg/kg
0.16 ± 0.015	0.25* ± 0.03	0.28* ± 0.04	0.275* ± 0.015

Mean ± SD alpha power (mV^2^/Hz x 10^-3^): F_(3, 32)_ = 40.45, p < 0.0001			
Control	Ketamine at a dose of:		
	10 mg/kg	20 mg/kg	30 mg/kg
0.16 ± 0.01	0.10* ± 0.01	0.10* ± 0.01	0.14*^# ± 0.01

Mean ± SD beta power (mV^2^/Hz x 10^-3^): F_(3, 32)_ = 31.55, p < 0.0001			
Control	Ketamine at a dose of:		
	10 mg/kg	20 mg/kg	30 mg/kg
0.15 ± 0.01	0.195* ± 0.03	0.22* ± 0.02	0.24*^ ± 0.02

Mean ± SD gamma power (mV^2^/Hz x 10^-3^): H = 27.28, p < 0.0001			
Control	Ketamine at a dose of:		
	10 mg/kg	20 mg/kg	30 mg/kg
0.01 ± 0.001	0.038 ± 0.008	0.05* ± 0.01	0.06*^ ± 0.01

^*^ *p* < 0.05 *vs*. control;

^^^ *p* < 0.05 *vs*. ketamine 10 mg/kg;

^#^ *p* < 0.05 *vs*. ketamine 20 mg/kg.

The decomposition of the spectral power distribution of the ketamine groups revealed a greater amplitude in the delta, theta, beta, and gamma waves in comparison with the control group. Significant differences were found in the delta waves (1–4 Hz) between the control group and the ketamine groups at the highest doses (20 mg/kg, *p* = 0.0212; 30 mg/kg, *p* < 0.0001). At the highest dose, the ketamine group also had a significantly greater mean spectral power in the delta band than the two lower doses (*p* < 0.001 in both cases; Figure 3B and Table 1).

In the case of the theta waves (4–8 Hz), the oscillation of all the ketamine treatment groups was significantly higher than that of the control group (*p* < 0.0001 in all cases) but did not vary significantly (*p* > 0.05) between doses (Figure 3C; Table 1). All the ketamine groups also presented a significant reduction (*p* < 0.01 for all comparisons) in the mean spectral power of the alpha wave frequency (8–12 Hz) in comparison with the control group. The alpha wave bandpower of the higher-dose (30 mg/kg) ketamine group was significantly higher than that of both lower doses (*p* < 0.001 in both cases; Figure 3D and Table 1).

Ketamine also provoked an increase in amplitude at the higher wavelengths. All three ketamine groups had a significantly higher amplitude (*p* < 0.001 in all three cases) in the beta wave bandpower (12–28 Hz), in comparison with the control group (Figure 3E; Table 1). The highest-dose group (30 mg/kg) also had a significantly higher amplitude (*p* = 0.0010) than the lowest-dose group (10 mg/kg). In the case of the gamma waves (28–40 Hz), the amplitude of the medium- (20 mg/kg; *p* = 0.0016) and high-dose (30 mg/kg; *p* < 0.0001) ketamine was significantly higher than that of the control group (Figure 3F; Table 1). The highest-dose group also had a significantly higher amplitude than the lowest-dose (10 mg/kg) group (*p* = 0.0356).

**FIGURE 4:**
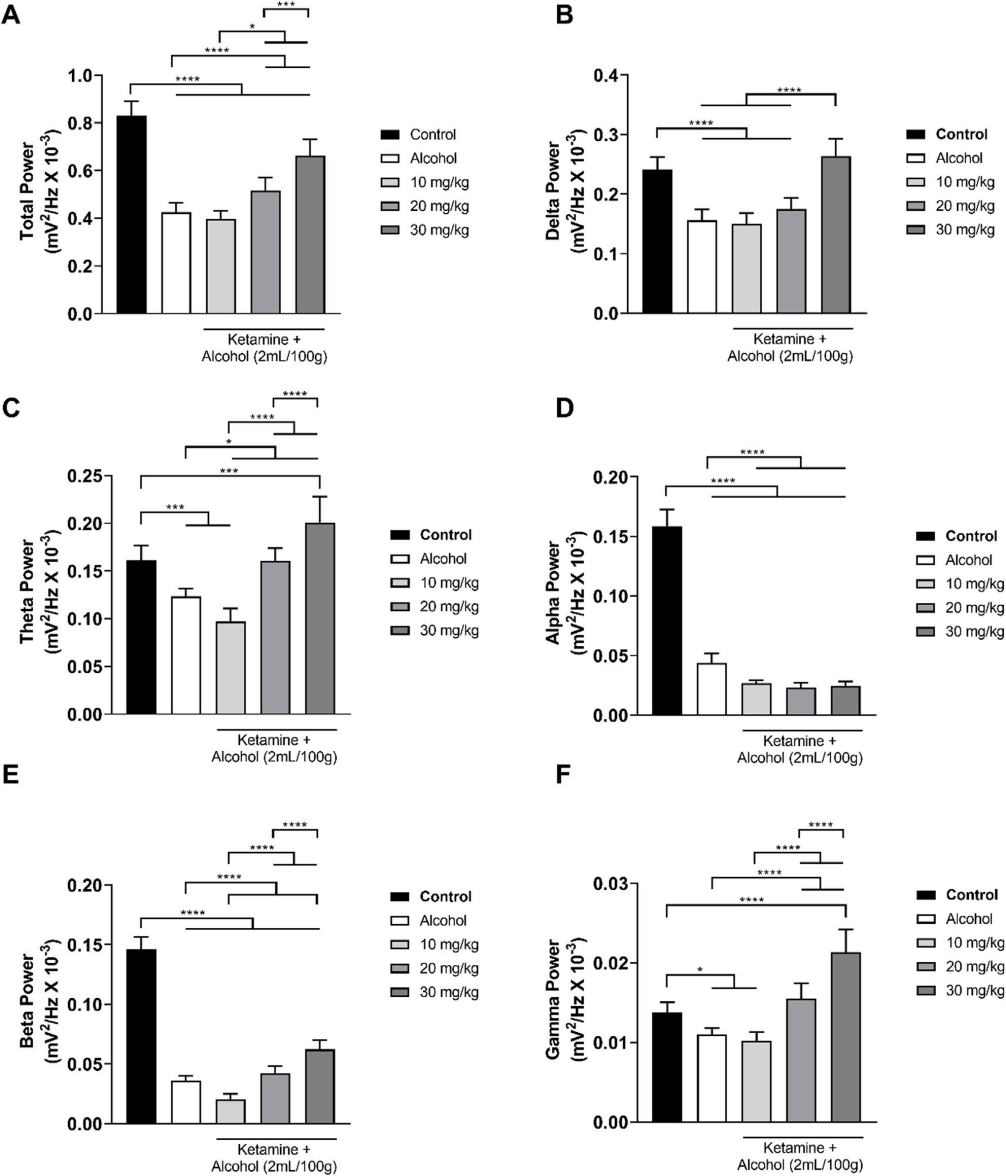
Linear distribution of the frequencies (mean ± SD) of the brainwaves of the animals in the alcohol, alcohol + ketamine and control groups (*n* = 9 animals/group): **(A)** Total bandpower; **(B)** Delta wave bandpower; **(C)** Theta wave bandpower; **(D)** Alpha wave bandpower; **(E)** Beta wave bandpower; **(F)** Gamma wave bandpower. All analyses are based on a one-way ANOVA with Tukey’s *post hoc* test. (*p < 0.05, ***p < 0.001, ****p < 0.0001).

In the treatments involving alcohol, the alcohol only group presented a reduction in both total wave power and the power of all the different frequencies (Figure 4). This pattern was further accentuated at the lowest dose of ketamine (10 mg/kg), in particular in the theta, alpha, and beta bandpowers. When associated with alcohol, however, the intermediate dose of ketamine (20 mg/kg) attenuated the reduction in brainwave power induced by alcohol on its own, with the theta and gamma brainwaves reaching those of the control group. At the highest dose of ketamine (30 mg/kg), the brainwave power was further attenuated, except in the case of the alpha band power. Overall, then, significant variation was found between the control group and all the other groups in the total power of the wave (*p* < 0.0001 in all cases), with a clear reduction in the total bandpower being observed in the group that received alcohol only in comparison with the control group (*p* < 0.0001). However, administration of ketamine at intermediate (20 mg/kg) and high doses (30 mg/kg) in association with alcohol reversed the effects (*p* < 0.0001) induced by alcohol alone (Figure 4A; Table 2).

**TABLE 2:** Electroencephalographic parameters of the total bandpower and the power of the delta, theta, alpha, beta, and gamma brainwaves in the animals exposed to alcohol and alcohol associated with different doses of ketamine.

Mean ± SD total power (mV^2^/Hz x 10^-3^): F_(4, 40)_ = 104.3, *p* < 0.0001				
Control	Alcohol	Alcohol + Ketamine at a dose of:		
		10 mg/kg	20 mg/kg	30 mg/kg
0.833 ± 0.06	0.43^*^ ± 0.04	0.396^*^ ± 0.03	0.515^*^#^ ± 0.055	0.66^*^#+^ ± 0.07

Mean ± SD delta power (mV^2^/Hz x 10^-3^): F_(4, 40)_ = 53.15, *p* < 0.0001				
Control	Alcohol	Alcohol + Ketamine at a dose of:		
		10 mg/kg	20 mg/kg	30 mg/kg
0.241 ± 0.021	0.16^*^ ± 0.02	0.15^*^ ± 0.02	0.175^*^ ± 0.02	0.26^^#+^ ± 0.03

Mean ± SD theta power (mV^2^/Hz x 10^-3^): F_(4, 40)_ = 50.64, *p* < 0.0001				
Control	Alcohol	Alcohol + Ketamine at a dose of:		
		10 mg/kg	20 mg/kg	30 mg/kg
0.16 ± 0.015	0.12^*^ ± 0.01	0.01^*^^ ± 0.01	0.16^^#^ ± 0.01	0.20^*^#+^ ± 0.03

Mean ± SD alpha power (mV^2^/Hz x 10^-3^): F_(4, 40)_ = 503.7, *p* < 0.0001				
Control	Alcohol	Alcohol + Ketamine at a dose of:		
		10 mg/kg	20 mg/kg	30 mg/kg
0.16 ± 0.01	0.04^*^ ± 0.01	0.03^*^^ ± 0.002	0.02^*^^ ± 0.004	0.02^*^^ ± 0.004

Mean ± SD beta power (mV^2^/Hz x 10^-3^): F_(4, 40)_ = 480.8, *p* < 0.0001				
Control	Alcohol	Alcohol + Ketamine at a dose of:		
		10 mg/kg	20 mg/kg	30 mg/kg
0.15 ± 0.01	0.035^*^ ± 0.004	0.02^*^^ ± 0.004	0.042^*#^ ± 0.005	0.06^*^#+^ ± 0.01

Mean ± SD gamma power (mV^2^/Hz x 10^-3^): F_(4, 40)_ = 58.18, *p* < 0.0001				
Control	Alcohol	Alcohol + Ketamine at a dose of:		
		10 mg/kg	20 mg/kg	30 mg/kg
0.013 ± 0.001	0.011^*^ ± 0.0008	0.010^*^ ± 0.001	0.015^^#^ ± 0.002	0.021^*^#+^ ± 0.003

^*^ *p* < 0.05 vs. control;

^^^ *p* < 0.05 vs. alcohol;

^#^ *p* < 0.05 vs. ketamine 10 mg/kg;

^+^ *p* < 0.05 vs. ketamine 20 mg/kg.

In the decomposition of the brainwaves, a significant (*p* < 0.0001 for all comparisons) reduction in the mean power of the delta wave was observed when comparing the control group with the alcohol only group and the low and intermediate dose (10, 20 mg/kg) alcohol + ketamine groups. However, the mean delta wave power of the alcohol + ketamine 30 mg/kg group was significantly greater than that of the other three alcohol treatment groups (*p* < 0.0001 in all cases), while it was also similar to that of the control group (*p* = 0.1181; Figure 4B and Table 2).

In the case of the theta waves, a reduction in the mean power was observed in the group that received alcohol only, in comparison with the control group (*p* = 0.0002). The alcohol + ketamine 10 mg/kg treatment further potentiated the effect observed in the alcohol only group (*p* = 0.0162), as well as being highly different (*p* < 0.0001) from the control group. The alcohol + ketamine 20 mg/kg group had significantly (*p* < 0.0001) higher theta wave power in comparison with the alcohol + ketamine 10 mg/kg group but was similar to the control group (*p* > 0.9999). The theta wave bandpower of the alcohol + ketamine 30 mg/kg group was significantly different from that of all the other alcohol treatments and was significant higher (*p* < 0.001) than that of the control group (Figure 4C; Table 2).

In the case of the alpha waves, there was a highly significant (*p* < 0.0001) reduction in the bandpower of all the alcohol treatment groups, in comparison with the control group. The alcohol + ketamine also further potentiated the effects of alcohol at all doses (*p* < 0.0001 for all comparisons; Figure 4D and Table 2).

As for the alpha waves, a highly significant (*p* < 0.0001) reduction in beta wave activity was recorded for all the alcohol treatment groups, in comparison with the control group. While the alcohol + ketamine 10 mg/kg treatment further potentiated the effects of alcohol alone (*p* = 0.0002), the alcohol + ketamine 30 mg/kg treatment resulted in a significant increase (*p* < 0.0001) in the power of the beta waves, in comparison with the alcohol only group, which means that the effects of the association of ketamine with alcohol are dose-dependent (Figure 4E; Table 2).

In the case of the gamma frequency oscillations, significant differences were recorded between the control and the alcohol (*p* = 0.0148), alcohol + ketamine 10 mg/kg (*p* = 0.0011), and alcohol + ketamine 30 mg/kg (*p* < 0.0001) groups. Once again, the higher alcohol + ketamine dose groups had significantly higher (*p* < 0.0001) gamma wave bandpower in comparison with the alcohol only treatment, indicating a dose-dependent pattern (Figure 4F; Table 2).

### 3.3 The association between ketamine and alcohol induces respiratory depression

**FIGURE 5:**
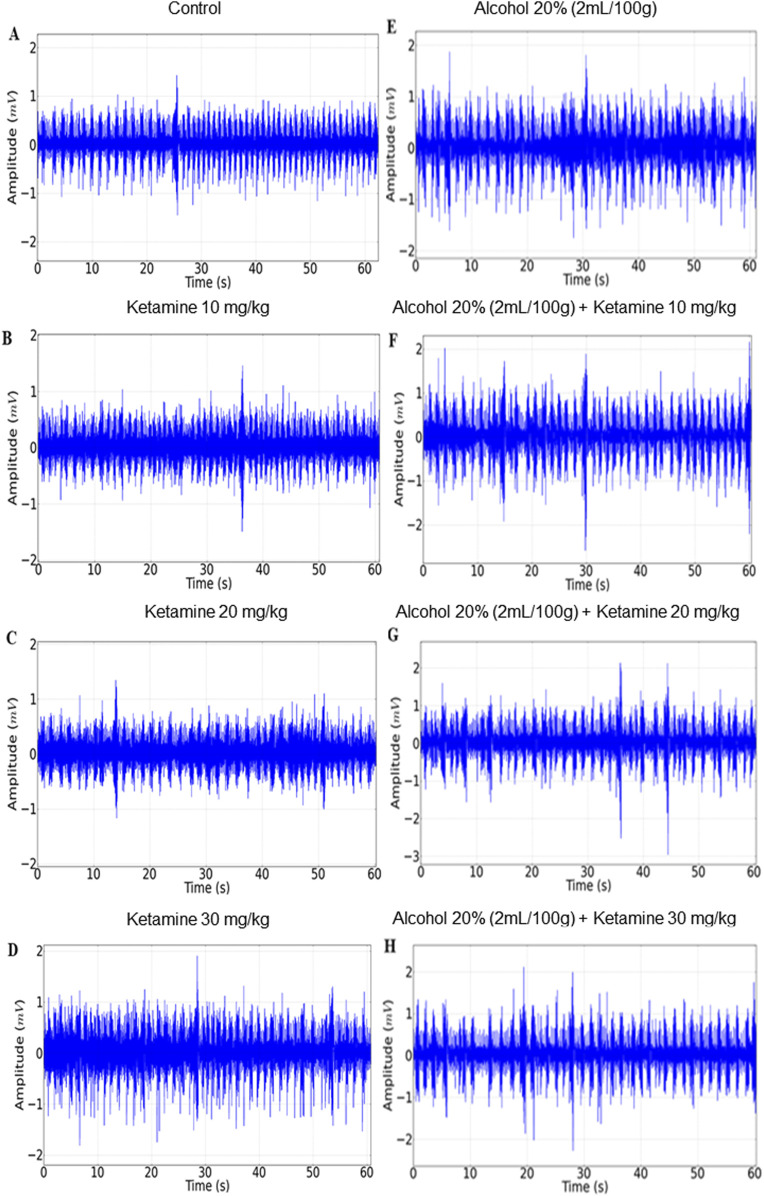
Representative 1-min electromyographic recordings of the intercostal muscle: **(A)** Control; **(B)** Ketamine 10 mg/kg; **(C)** Ketamine 20 mg/kg; **(D)** Ketamine 30 mg/kg; **(E)** Alcohol; **(F)** Alcohol + ketamine 10 mg/kg; **(G)** Alcohol + ketamine 20 mg/kg; **(H)** Alcohol + ketamine 30 mg/kg.

The activity of the intercostal muscle varied considerably among the different treatment groups, with a progressive decrease in respiratory activity being observed in the EMG traces with increasing doses of ketamine, in comparison with the control group. A more intense reduction was observed in the group that received alcohol only, with the association between alcohol and ketamine further potentiating the respiratory depression in the study animals (Figure 5).

**FIGURE 6:**
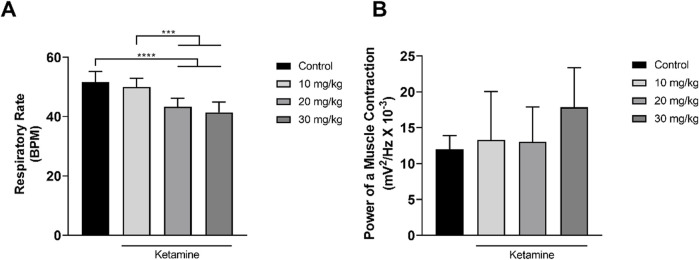
Variation in the mean ± SD respiratory function of the rats treated with varying doses of ketamine (n = 9 animals/group): **(A)** Respiratory rate (BPM = breaths per minute), by one-way ANOVA with Tukey’s *post hoc* test; **(B)** Power of contraction of the intercostal muscle, by Kruskal–Wallis test with Dunn’s *post hoc* test. (*p < 0.05, **p < 0.01, ***p < 0.001 and ****p < 0.0001).

Only the higher doses of ketamine (20, 30 mg/kg) induced a significant reduction in the respiratory rate in comparison with the control group (*p* < 0.0001; Figure 6A and Table 3). No variation was observed among the groups, however in the power of the contraction of the intercostal muscle (*p* > 0.05; Figure 6B and Table 3).

**TABLE 3:** Electromyographic parameters of the respiratory rate and the muscle contraction in the control and animals treated with varying doses of ketamine.

Mean ± SD respiratory rate (breaths per minute): F_(3, 32)_ = 20.93, *p* < 0.0001			
*Control*	*Ketamine at a dose of:*		
	*10 mg/kg*	*20 mg/kg*	*30 mg/kg*
51.56 ± 3.68	50.00 ± 2.96	43.33^*^^ ± 2.82	41.44^*^^ ± 3.43

Mean ± SD power of the muscle contraction (mV^2^/Hz x 10 ^-3^): H = 6.689, p = 0.0825			
Control	Ketamine at a dose of:		
	10 mg/kg	20 mg/kg	30 mg/kg
12.04 ± 1.90	13.29 ± 6.75	13.04 ± 4.83	17.85 ± 5.51

^*^ *p* < 0.05 *vs*. control;

^^^ *p* < 0.05 *vs*. ketamine 10 mg/kg.

**FIGURE 7:**
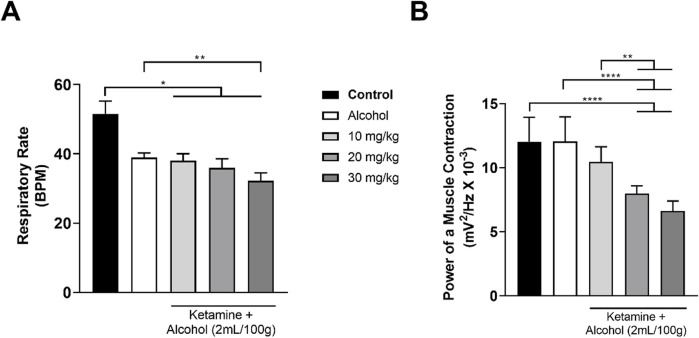
Variation in the mean ± SD respiratory function of the rats treated with alcohol only and alcohol + varying doses of ketamine (n = 9 animals/group): **(A)** Respiratory rate (BPM = breaths per minute), one-way ANOVA with Tukey’s test: *p < 0.05, **p < 0.01; **(B)** Power of contraction of the intercostal muscle, Kruskal–Wallis test with Dunn’s *post hoc* test: **p < 0.01, ****p < 0.0001.

Both alcohol only and alcohol + ketamine at higher doses (20, 30 mg/kg) induced significant respiratory depression, in comparison with the control group (*p* < 0.05 in all cases; Figure 7A and Table 4). However, only the two higher-dose alcohol + ketamine groups presented a significant reduction in the contraction of the intercostal muscle in comparison with the control group (*p* < 0.0001 in both cases), as well as in comparison with the alcohol only (*p* < 0.0001) and the alcohol + ketamine 10 mg/kg groups (*p* < 0.01; Figure 7B and Table 4).

**TABLE 4:** Electromyographic parameters of the respiratory rate and the muscle contraction in the animals treated with alcohol alone and alcohol + varying doses of ketamine.

Mean ± SD respiratory rate (breaths per minute): H = 35.23, *p* < 0.0001				
*Control*	*Alcohol*	*Alcohol + Ketamine at a dose of:*		
		*10 mg/kg*	*20 mg/kg*	*30 mg/kg*
51.56 ± 3.68	38.89 ± 1.36	38.00^*^ ± 2.00	35.90^*^ ± 2.67	32.22^*^^ ± 2.28

Mean ± SD power of the muscle contraction (mV^2^/Hz x 10^-3^): F_(4, 40)_ = 27.91, p < 0.0001				
Control	Alcohol	Alcohol + Ketamine at a dose of:		
		10 mg/kg	20 mg/kg	30 mg/kg
12.04 ± 1.90	12.04 ± 1.93	10.45 ± 1.19	8.00^*^#^ ± 0.61	6.62^*^#^ ± 0.78

^*^ *p* < 0.05 *vs*. control;

^^^ *p* < 0.05 *vs*. alcohol;

^#^ *p* < 0.05 *vs*. ketamine 10 mg/kg.

## 4 Discussion

Given the ongoing growth in the number of persons consuming alcohol in combination with ketamine, worldwide, it is increasingly important to understand the changes in brain connections caused by this practice. The concomitant use of alcohol and ketamine is characteristic of the party culture, where at least 65% of the adolescents and young adults that abuse ketamine also consume alcohol (Curran and Morgan, 2000; Morgan et al., 2009; Morgan et al., 2010). The interval between late adolescence and early adulthood is marked by a range of shifts, not only in the body, but also in attitudes and social relationships, and in particular, the need for peer acceptance. This developmental period is associated with impulsivity, reward-sensitivity, and sensation-seeking behaviors (Urošević et al., 2012; Harden and Tucker-Drob, 2011), which often reinforce risky habits, such as unprotected sex and the consumption of narcotics, including alcohol and ketamine (Marco et al., 2022; Cohen et al., 2022; Rehm et al., 2009). Exposure to drugs during this developmental stage may lead to maladaptive changes in the brain that can result in disorders of mental health, including addiction (Kehinde et al., 2019).

Ketamine is an N-methyl-D-aspartate (NMDA) receptor antagonist that has widespread effects on brain activity. Previous studies have shown that it may also act through other non-glutamatergic pathways to induce rapid changes in a broad class of “neurotic disorders”, even after a single dose (Averill et al., 2017; Glue et al., 2017). In healthy participants, a single dose of ketamine can reduce the bandpower of the low-frequency (delta and theta) waves (Hong et al., 2010; de la Salle et al., 2016), while increasing the bandpower of higher frequency waves, such as the gamma waves (>28 Hz). Ketamine may also increase the theta power, while simultaneously decreasing the alpha power, in particular in the pre-frontal cortex (PFC), a pattern also found in patients with chronic schizophrenia (Kim et al., 2015), which may be related to the dissociative properties of the drug. The changes in bandpower may be interspersed, as observed in the decrease in the delta, alpha, and beta waves associated with an increase in the theta and gamma waves (Muthukumaraswamy et al., 2015; Rivolta et al., 2015). The alterations in the brain activity patterns measured by the quantified EEG (qEEG) may be related to the dose of ketamine and the associated substances (Shadli et al., 2018).

The results of the present study indicate clearly that the application of ketamine increases delta bandpower in a dose-dependent manner. This increase in delta bandpower was also associated with an increase in the theta and a reduction in the alpha waves. These findings are consistent with those of Ching and Brown (2014) and Purdon et al. (Purdon et al., 2013), who showed that subanesthetic doses of ketamine promote an increase in slow wave power, with a concomitant reduction in alpha bandpower. This qEEG pattern is typical of light sleep (Purdon et al., 2013), which suggests that subanesthetic doses, even when reported as having a predominantly analgesic effect (Cohen et al., 2022; Balzer et al., 2021), may provoke varying degrees of sedation.

However, the administration of ketamine may result in alternative patterns. For example, de la Salle et al. (2016) recorded an increase in high-frequency (gamma) qEEG activity, with no concomitant increase in the low frequency (delta and theta) waves in healthy, resting-state individuals treated with a sub-anesthetic dose of ketamine, which was related to perceptual/dissociative symptoms. These findings contrast with those of the present study, which recorded a combined increase in these waves. These discrepancies would be expected, given that different populations are likely to vary in their response, depending on the dosage and age at which the ketamine was administered (Hong et al., 2010; Parrino et al., 2014).

The results of the present study are consistent with the findings of Muthukumaraswamy et al. (2015), who observed comparable changes in the frequency pattern after the administration of ketamine, with an increase in the theta (slow) waves and overall gamma activity (fast waves). This association of an increase in the slow waves with a high amplitude and fast waves with a low amplitude generates a pattern of desynchronization in the EEG (Parrino et al., 2014; Simor et al., 2016), which is known as an alternating cyclical pattern, given that the gamma waves may increase and decrease in bandpower intermittently (Terzano et al., 2001). These behavioral patterns are related to a reduction in the state of vigilance (Ferri et al., 2007). Oscillations in the theta waves are typical of drowsiness and relaxed wakefulness (Matousek and Petersén, 1983; Ulett and Itil, 1969), whereas high delta and theta coherence are related to lower levels of consciousness (Fingelkurts et al., 2012; Leon-Carrion et al., 2008). Taken together, these findings indicate that ketamine reduces arousal.

The reduction of vigilance caused by the ketamine and the appearance of states of cortical desynchronization in the brain indicate a state of cerebral hyperexcitation. This interaction is observed primarily between the neurons of the PFC and the parietal cortex, an interaction that may account for the dissociative and hallucinogenic effects of ketamine, depending on the degree of hyperexcitation (Kaiser et al., 2015).

Some authors have emphasized the importance of low frequency waves during sleep, and in particular, that the variation in the bandwidth of these waves can be interpreted as a measure of the depth of sleep (Terzano et al., 2001). The Brain-Derived Neurotrophic Factor (BDNF), which is a peripheral marker of neuronal plasticity, is produced intensively during sleep, and while ketamine was associated with high levels of delta and theta bandpower in the present study, it is not possible to confirm conclusively that the production of BDNF increases when ketamine is used, given that the increase in low frequency waves observed here was accompanied by an increase in the gamma waves, which suggests the occurrence of anomalies in brain connections (Duncan et al., 2013).

This desynchronization is not permanent. Scheidegger et al. (2012) and Bonhomme et al. (2016) observed a decrease in the functional connectivity of the PFC, as measured by qEEG, and that this phenomenon will typically normalize in up to 1 hour after the application of the ketamine (0.5 mg/kg), and then decrease again after 24 h, which may, in turn, be associated with greater changes in the glutamine/glutamate levels of the perigenual anterior cingulate cortex (Li et al., 2020). However, some research has shown that the depth of the anesthesia induced by ketamine is related negatively to the cortico-cortical connectivity of the PFC (Bonhomme et al., 2016).

Alcohol is also widely consumed and, due to its low molecular weight, it can be distributed widely in many different types of bodily tissue, in particular, the brain (Albanese, 2012; Crews et al., 2016). Many previous studies have recorded the neurotoxic effects of alcohol, which lead primarily to neuronal death, abnormalities in synaptic plasticity, cognitive dysfunctions, and cortical atrophy (Oliveira et al., 2015; Guerri and Pascual, 2010; Witt, 2010). While alcohol and ketamine may often be consumed together, few studies have focused on their combined toxic effects in the brain. The present study is the first to describe the changes in brain waves associated with the combination of ketamine and alcohol.

A number of previous EEG studies have determined the effects of alcohol on brainwaves. Saletu-Zyhlarz et al. (2004) recorded a decrease in the delta and alpha bandpower following the consumption of alcohol, which may reflect a deficit in attention and the capacity of the individual to retrieve information from their memory. The reduction in alpha and delta bandpower recorded in the present study is consistent with these findings. Some studies have nevertheless identified a reduction in beta wave activity and an increase in alpha wave activity in young adults exposed to moderate doses of alcohol, and that the increase in alpha activity caused by the alcohol varies depending on the brain region analyzed (Van Reen et al., 2006). This variation may also be related to the desynchronization of the neurons located in the vicinity of the electrode (somatosensory cortex), leading to a decrease in signaling, which may cause the individual to experience temporary loss of sensitivity or proprioception (Andrew and Fein, 2010).

The combined use of ketamine and alcohol may have synergic effects, with potentially harmful consequences, given the capacity of both substances to modulate glutamatergic and GABAergic pathways, primarily through a reduction in glutamatergic activity in the NMDA receptors (Strong and Kabbaj, 2020). Depending on the frequency and level of use, these synergic effects may provoke a wide range of symptoms, which may be reflected in the variation in the EEG recordings of the different concentrations of ketamine administered in association with alcohol. This would produce alterations in the brainwave patterns that do not necessarily follow a systematic pattern, reflecting possible synergism by potentiation.

The results of the present study indicated a reduction in delta bandpower activity following the ingestion of alcohol alone or in combination with ketamine, but with no synergistic effects or potentiation at the lower doses of ketamine (10, 20 mg/kg). The highest dose (30 mg/kg) nevertheless resulted in an increase in the delta bandpower to close to the control value, although this cannot be interpreted as a return to the baseline state, given the presence of two pharmacological agents in the body, but rather, an increase in inhibition (Saletu-Zyhlarz et al., 2004). This confirms that the association of alcohol and ketamine can have negative repercussions for high-level functions, such as cognition and memory (Jones et al., 2006).

Like the low frequency waves, a reduction in the alpha wave bandpower was also observed in the animals that received alcohol, an effect that was enhanced significantly by ketamine, irrespective of the dose. In contrast with the present study, which recorded a reduction in alpha bandpower 5 minutes after the administration of the alcohol, Lukas et al. (Lukas et al., 1986) reported an increase in spontaneous alpha activity 1 hour after the consumption of 0.695 g/kg of alcohol by volunteers, while Ehlers et al. (1989) recorded a reduction in the potency of the alpha waves 90 min after the consumption of a dose of 0.75 mg/kg. This indicates that alcohol may have both inhibitory and excitatory effects over time, depending on the dose. Other studies have shown that the reduction in alpha waves, which is related to the loss of the capacity for attention and wakefulness, is also associated with failures in the visualization and execution of motor processes, as well as interfering with the connection between the occipital and somatomotor cortexes (Athanasiou et al., 2018; Sabate et al., 2011). This suggests that alcohol interferes with both proprioception and locomotion, and that the association with ketamine increases this effect, with the joint use of these two substances being an even greater danger, due to the increased risk of traffic accidents and falls.

While the potency of the beta waves is diminished considerably by alcohol, it is increased by ketamine in a dose-dependent manner. There is no conclusive evidence on the effects of these drugs on beta waves, although Akeju et al. (2016) recorded a reduction in the alpha/beta patterns under pure ketamine, which contrasts with the findings of the present study, although this difference may be a consequence of the distinct analytical strategies employed for the interpretation of ketamine-induced anesthesia.

In contrast with the exclusive exposure to ketamine, there was a reduction in the bandpower of the gamma wave in the animals exposed to alcohol. This phenomenon was reversed, however, at higher doses of ketamine, which does not mean a return to the basal state, but rather, that the use of ketamine induced hyperexcitation of the neurons that had been inhibited by the alcohol (Parrino et al., 2014; Simor et al., 2016; Kaiser et al., 2015). These oscillations in gamma bandpower, which were observed primarily in the pyramidal neurons containing GABA receptors that receive the input of parvalbumin-positive interneurons (caused by the blockade of NMDA receptors), reflect cortical hyperactivity, observed in the animals that received ketamine only (Hakami et al., 2009; Lee et al., 2003a; Sullivan et al., 2015). Increased gamma bandpower is also characteristic of patients with schizophrenia, due to the diminished activity of the parvalbumin-containing GABAergic inhibitory interneurons, which are subject to NMDAR modulation from the pyramidal cells (Lee et al., 2003b). In this context, the administration of higher doses of ketamine together with alcohol may be reflected in abnormal hyperactivity of the neuronal circuits, correlated with behavioral and cognitive impairments (Abdallah et al., 2018), including those found in schizophrenia.

The effects of ketamine on the CNS are contentious, given that there is considerable evidence of the potential of this drug for the treatment of resistant depression, alcoholism, status epilepticus, and pain syndromes (Pribish et al., 2020). Clinical studies have shown that psychotherapy and therapeutic doses of ketamine can have synergistic effects on abstinence rates in the treatment of patients with disorders of alcohol abuse (Das et al., 2019; Dakwar et al., 2020; Rothberg et al., 2021). Some studies have also shown that the therapeutic benefits of ketamine are related to its anti-depressant effects. For example, Duncan et al. (2013) showed that the administration of ketamine enhanced slow wave sleep (measured as the delta sleep ratio), which results in an improvement in the depressive symptoms of patients with major depressive disorder, and is also an indicator of plasticity, based on the levels of BDNF (Duncan et al., 2013). These findings indicate that ketamine works through a “psychoplastogen” mechanism by enhancing neuroplasticity and synaptogenesis and promotes a therapeutic window of time during which psychotherapeutic interventions for alcoholism may be more effective (Kelson et al., 2023). However, further research will be necessary to establish a more effective balance between the therapeutic use of ketamine and addiction.

**FIGURE 8:**
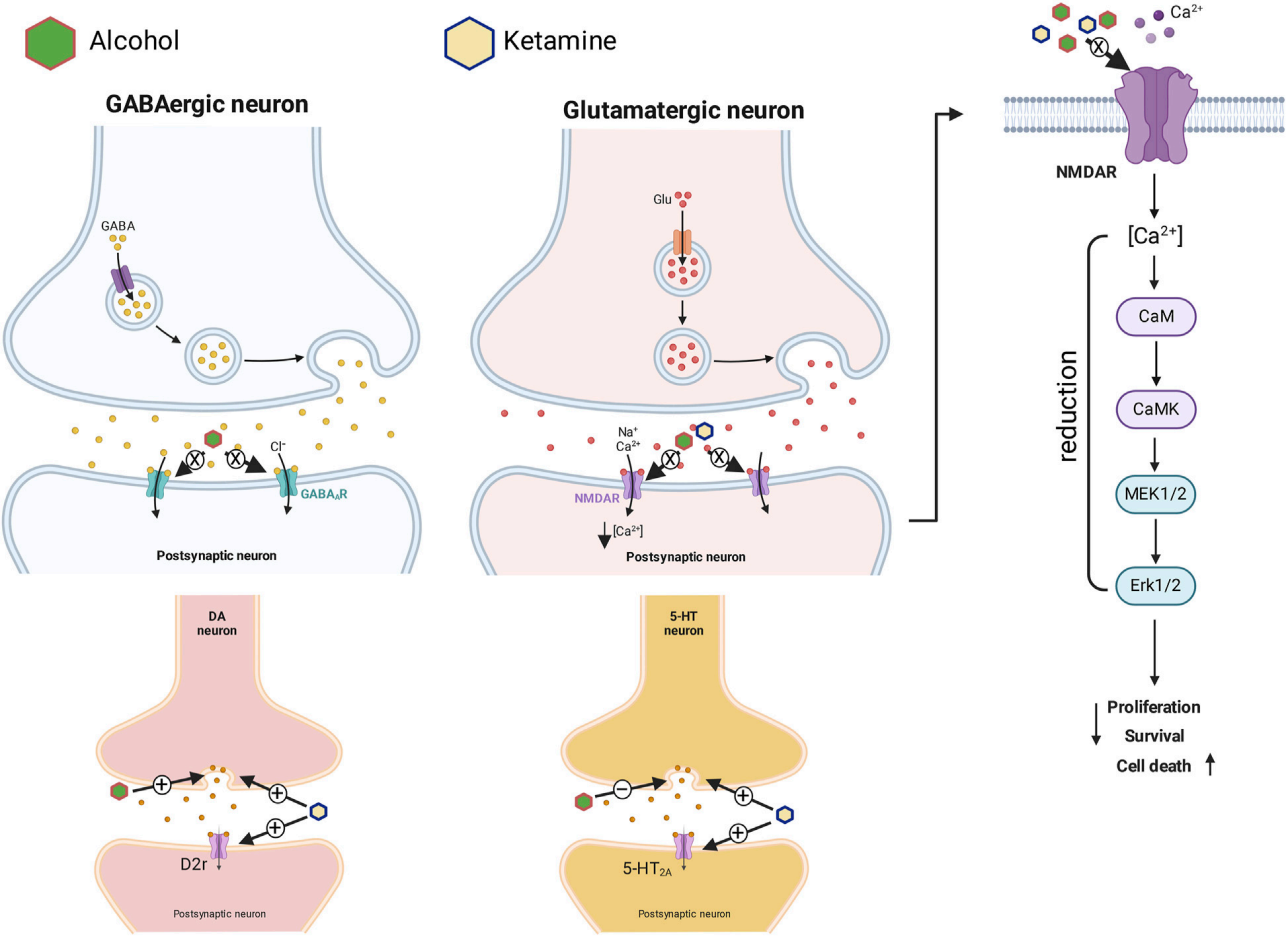
Pharmacodynamics of the action of ketamine in association with alcohol on GABAergic and glutamatergic synapses (with intracellular signaling on the right), and dopaminergic and serotoninergic synapses. + = Activation, elevation or potentiation; − = inhibition or reduction; X = blocking.

Ketamine can also cause hyperfunction of the D2-type dopamine receptor, which contributes intensively to hallucinogenic symptoms, in either acute or chronic exposure (Vollenweider et al., 2000). A ketamine-induced dissociative state evokes a range of symptoms, behaviors, and cognitive deficits that are similar to a psychosis (Ballard and Zarate, 2020). Some studies in animal models (Martin et al., 1998; Pal et al., 2015) have in fact shown that the NMDA blockers can cause an increase in the release of neurotransmitters–not only glutamate and dopamine, but also serotonin and acetylcholine–in the prefrontal cortex. The release of these neurotransmitters into the synaptic cleft under the influence of alcohol and ketamine can induce hallucinations and even cell death. This may be caused by the hyperactivation of the dopaminergic circuit and the reduction of serotonin in the brain when blood concentrations of alcohol and ketamine are high (Figure 8). It thus seems likely that the overlapping molecular toxicological mechanisms that are activated by the association of alcohol and ketamine may contribute synergistically to the suppression of brain wave activity observed in the EEG.

Overall, then, this investigation of the repercussions of the associated use of alcohol and ketamine on cortical electrical activity provides important insights into the potential damage that these substances can cause to the CNS. These findings are especially relevant due to the increasing combined recreational use of these two substances in populations around the world. While this study revealed synergistic effects, the depressant effect on the CNS is attributed primarily to the use of alcohol, whereas ketamine induces psychotomimetic-like patterns in the brainwaves, with increasing slow and gamma waves, ranging in frequencies of up to 40 Hz. These findings contribute to a better understanding of the effects of the combination of alcohol and ketamine on the nervous system, and the potential complications that their frequent abuse can cause. This will support research for the development of more effective management protocols for the prevention of the harm caused by the use of these drugs and the treatment of addiction.

## Data Availability

The raw data supporting the conclusions of this article will be made available by the authors, without undue reservation.
